# Heritability of skewed X-inactivation in female twins is tissue-specific and associated with age

**DOI:** 10.1038/s41467-019-13340-w

**Published:** 2019-11-25

**Authors:** Antonino Zito, Matthew N. Davies, Pei-Chien Tsai, Susanna Roberts, Rosa Andres-Ejarque, Stefano Nardone, Jordana T. Bell, Chloe C. Y. Wong, Kerrin S. Small

**Affiliations:** 1 Department of Twin Research & Genetic Epidemiology, King’s College London, London, SE1 7EH UK; 2 Ervaxx Limited, 71 Kingsway, London, WC2B 6ST UK; 3 Department of Biomedical Sciences, Chang Gung University, Taoyuan, Taiwan; 4 Genomic Medicine Research Core Laboratory, Chang Gung Memorial Hospital, Linkou, Taiwan; 5 Social, Genetic & Developmental Psychiatry Centre, Institute of Psychiatry, Psychology & Neuroscience, King’s College London, London, SE5 8AF UK; 6 St John’s Institute of Dermatology, Faculty of Life Science & Medicine, King’s College London, London, SE1 9RT UK; 7 Division of Endocrinology, Diabetes, and Metabolism, Department of Medicine, Beth Israel Deaconess Medical Center, Harvard Medical School, Boston, MA 02215 USA

**Keywords:** Gene expression, Chromosomes, Dosage compensation, Rheumatoid arthritis

## Abstract

Female somatic X-chromosome inactivation (XCI) balances the X-linked transcriptional dosages between the sexes. Skewed XCI toward one parental X has been observed in several complex human traits, but the extent to which genetics and environment influence skewed XCI is largely unexplored. To address this, we quantify XCI-skew in multiple tissues and immune cell types in a twin cohort. Within an individual, XCI-skew differs between blood, fat and skin tissue, but is shared across immune cell types. XCI skew increases with age in blood, but not other tissues, and is associated with smoking. XCI-skew is increased in twins with Rheumatoid Arthritis compared to unaffected identical co-twins. XCI-skew is heritable in blood of females >55 years old (h^2^ = 0.34), but not in younger individuals or other tissues. This results in a Gene x Age interaction that shifts the functional dosage of all X-linked heterozygous loci in a tissue-restricted manner.

## Abstract (web summary)

Skewing of X chromosome inactivation (XCI) occurs when the silencing of one parental X chromosome is non-random. Here, Zito et al. report XCI patterns in lymphoblastoid cell lines, blood, subcutaneous adipose tissue samples and skin samples of monozygotic and dizygotic twins and find XCI skew to associate with tissue and age.

## Introduction

To balance the X-linked transcriptional dosages between the single X chromosome of males and the two X chromosomes of females, one X chromosome is silenced in female placental mammals^1^. The X-chromosome inactivation (XCI) process starts during preimplantation phases of human embryonic development, presumably at around the eight-cell stage^2^. XCI is initiated by the transcription of *XIST*, a 17 kb, alternatively spliced long noncoding RNA mapped to Xq13.2 and exclusively expressed on the inactive X (Xi)^3^. Once transcribed, *XIST* molecules spread in *cis* along the X chromosome^4,5^ inducing a progressive epigenetic silencing through the recruitment of chromatin remodeling enzymatic complexes, which impose repressive histone and DNA changes on the Xi chromosome^6,7^. Within each cell, the parental X chromosome selected for inactivation seems to occur at random, and the Xi is mitotically inherited to future somatic daughter cells. This random inactivation results in a mosaic of cells within an individual, where overall, a balanced expression (50:50) of both parental X-linked alleles is expected. Asymmetric selection of the X chromosome to inactivate causes the predominance of one parental Xi in a population of cells, unbalancing the X-linked transcriptional and allelic dosages toward one parental X chromosome. This phenomenon, known as skewed XCI (or nonrandom XCI), occurs when at least 80% of cells within a tissue inactivate the same parental X chromosome. The factors underlying primary skewed XCI are varied and several mechanisms are possible^8^. Secondary (or acquired) skewed XCI can result from positive selection of cells that after having inactivated a particular parental X, acquire a survival advantage over cells who inactivated the other parental X chromosome. Skewed XCI patterns can also be generated by the stochastic overrepresentation of cell clones in a given tissue, due for instance, to depletion of stem cell populations.

Comprised of 155 MB and containing >800 protein-coding genes, the X chromosome represents approximately the 5% of the haploid human genome. In heterozygous females with skewed XCI, the X-linked transcriptional and allelic dosages of silenced genes are unbalanced and may be functionally homozygous. Skewed XCI is a major cause of discontinuity of dominance and recessiveness, as well as penetrance and expressivity of X-linked traits. How skewed XCI patterns modulate phenotypes in females, and whether they are a cause or a consequence of associated phenotypes is not fully understood. Skewed XCI patterns have been observed in females with X-linked diseases^9–11^, autoimmune disorders^12,13^, as well as in breast^14^ and ovarian cancer^15^. In autoimmune diseases with higher prevalence in females, including rheumatoid arthritis (RA) and systemic lupus erythematosus, XCI is hypothesized to play a role. Chromosome X is enriched for immune-related genes and skewed XCI patterns could cause the breakdown of thymic tolerance induction processes^16^ conferring a high predisposition to develop autoimmunity (reviewed in^17^). XCI skewing levels in blood tissues have been associated with ageing, with multiple studies indicating an increase after 50–60 years of age^18–23^. To date, the mechanisms underlying skewed XCI in humans are not fully understood. Several twin studies have reported that genetic factors contribute to XCI skewing in blood-derived cells^22,24^, while other evidence indicated that most of the XCI skewing levels in human are acquired secondarily^25^.

Nearly all studies of XCI skewing levels in humans have been carried out in peripheral blood samples or in very small sample sizes^26^, while XCI patterns in other tissues have not been studied in great detail^20,27,28^. In this study, we comprehensively assess XCI patterns in a multi-tissue sample of nearly 800 female twins from the TwinsUK cohort^29^. We quantify the degree of skewing of XCI using a metric based on *XIST* allele-specific expression (ASE) from paired RNA-seq and DNA-seq data in four tissues (LCLs, whole-blood, fat and skin) and in multiple immune cell types (monocytes, B-cells, T-CD4^+^, T-CD8^+^, NK) purified from two identical co-twins. We examine the tissue-specific prevalence of skewed XCI patterns, compare the XCI skewing levels between tissues and across immune cell types within the same individual, and evaluate the association between XCI skewing and age, a complex autoimmune disease and lifestyle (smoking) traits. We show that XCI patterns are highly tissue-specific and shared across immune cell types within an individual, and that XCI skew in haematopoetic tissues increases with age. We investigate the factors underlying the skewed XCI using classical twin models to characterize the extent of the influence of genetic and environmental factors on the tissue-specific skewed XCI. We show that heritability of XCI skew is restricted to blood tissues of females >55 years old (*h*^2^ = 0.34), indicating that XCI patterns have both a heritable and environmental (age) basis.

## Results

### Quantification of degree of XCI skewing in TwinsUK

We assessed XCI patterns in multi-tissue samples from female twin volunteers from the TwinsUK cohort aged 38–85 years old (median age = 60; Supplementary Fig. 1)^29,30^. We quantified the degree of skewing of XCI using a metric based on *XIST* ASE from paired RNA-seq and DNA-seq data. *XIST* is uniquely expressed from the Xi^3,28,31^, so the relative expression of parental alleles within the *XIST* transcript are representative of XCI skewing levels in a bulk sample. Skewed XCI patterns can be detected and quantified from the expression levels of *XIST*-linked heterozygous variants^32^. Furthermore, transcriptional assays based on single monoallelically expressed X-linked genes, like *XIST*, have been used as a complement to the HUMARA assay to quantify XCI patterns^25,33^. We have also calculated the XCI patterns using an alternative method, based on the ASE of all non-pseudo-autosomal region (PAR) heterozygous loci available in a sample^34^ as done in supplements. We ran a series of benchmarking analyses to compare the non-PAR_ASE_ calls to the *XIST*_ASE_-based XCI skew calls (Supplementary Note 1, Supplementary Figs. 2–4) and demonstrated that the *XIST*_ASE_ is the appropriate method to use in our analyses. RNA-seq and genotype data were available for 814 lymphoblastoid cell lines (LCL) samples, 395 whole-blood samples, 766 subcutaneous adipose tissue samples (herein referred as fat) and 716 skin samples. After stringent quality control, we obtained *XIST*_ASE_ calls for 422 LCL samples, 72 whole-blood samples, 378 fat samples and 336 skin samples. The smaller sample size for whole-blood was due to the relatively smaller size of the starting dataset and the relatively lower RNA-seq coverage of this tissue in our dataset. In order to have an absolute measure of the magnitude of the XCI skewing levels in each sample, we calculated the degree of skewing of XCI (DS) from the *XIST*_ASE_ calls. DS is defined as the absolute deviation of the *XIST*_ASE_ from 0.5 (see “Methods”) and it has been similarly used in other investigations to assess XCI patterns^21,35,36^ and the XCI status of X-linked genes^37^. In line with previous investigations^22,38^ we classified samples with DS < 0.3 (corresponding to 0.2 < *XIST*_ASE_ < 0.8) to have random XCI, and samples with DS ≥ 0.3 (corresponding to *XIST*_ASE_ ≤ 0.2 or *XIST*_ASE_ ≥ 0.8) to have skewed XCI. Unless otherwise specified, DS was used in all the analyses performed.

**Fig. 1:**
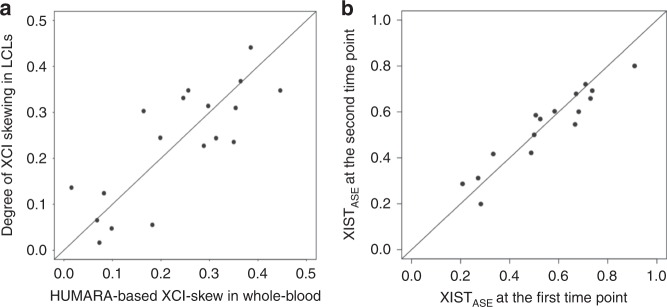
*XIST*_ASE_ is a reproducible and accurate proxy of XCI skewing levels. **a** Scatter plot showing the comparison between the XCI skewing levels in *n* = 18 whole-blood and LCLs samples quantified with HUMARA and *XIST*_ASE_ respectively. **b** Scatter plot of the *XIST*_ASE_ at time point 1 and at time point 2 in *n* = 16 whole-blood samples. Diagonal line indicates perfect concordance in the data (*ρ* = 1). HUMARA human androgen receptor assay.

We assessed the robustness of our estimates of the degree of skewing with an alternative DNA-based measure of XCI, the human androgen receptor assay (HUMARA)^39^. HUMARA was and is still the “gold standard” technique to assess XCI patterns. A previous study has reported good replicability between HUMARA and expression-based quantification of XCI skewing^40^. We used HUMARA to measure XCI skewing levels in 18 archived whole-blood DNA samples obtained at the same clinical visit as the LCLs samples. Spearman’s correlation between the quantifications was 0.8 (*P* = 7 × 10^−5^) revealing a high degree of reproducibility between the both *XIST*_ASE_ and HUMARA methods and the LCLs and whole blood (Fig. 1a).

Previous investigations have reported that the XCI skewing levels increase with age in blood tissues, as discussed below. While it would be expected that increases in XCI skewing levels would be observed over relatively large time spans, we would expect minimal variations of XCI skewing levels between two close time points. We therefore reasoned that the sensitivity of our quantifications could also be assessed by comparing the XCI skewing levels in the same individuals at close time points. Briefly, using a publicly-available longitudinal whole-blood RNA-seq dataset from the TwinsUK cohort^41^, we generated *XIST*_ASE_ calls at two time points (1–2.7 years later) in 16 samples (see “Methods”). The Spearman’s correlation between the *XIST*_ASE_ calls at the first time point and the *XIST*_ASE_ calls at the second time point was 0.94 at *P* < 2 × 10^−15^ (Fig. 1b). This indicates that *XIST*_ASE_ is a sensitive proxy when assessing the stability of XCI patterns over short time periods. Overall, these results indicate that *XIST*_ASE_ is a reproducible, accurate and sensitive proxy of XCI skewing levels.

### Skewed XCI is tissue-specific with higher prevalence in blood-derived tissues

**Fig. 2:**
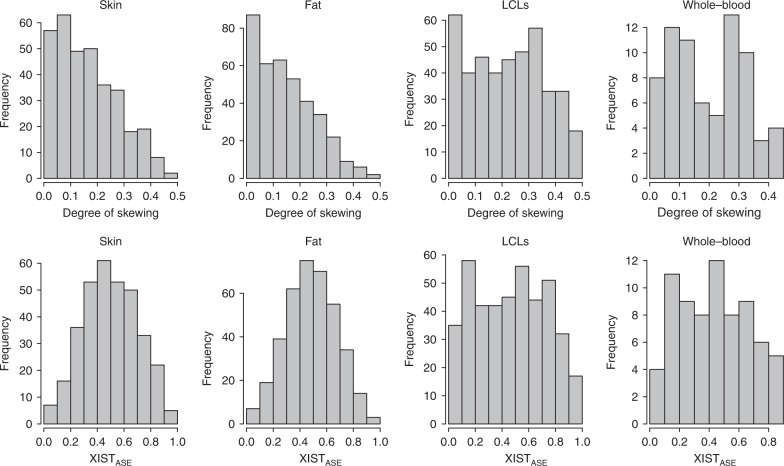
Skewed X-inactivation varies across tissues with a higher prevalence in blood than fat and skin tissues. Distribution of the degree of skewing (top row) and *XIST*_ASE_ (bottom row) in LCLs (*n* = 422 samples), whole-blood (*n* = 72 samples), fat (*n* = 378 samples) and skin (*n* = 336 samples) tissues. *XIST*_ASE_ *XIST* allele-specific expression.

**Fig. 3:**
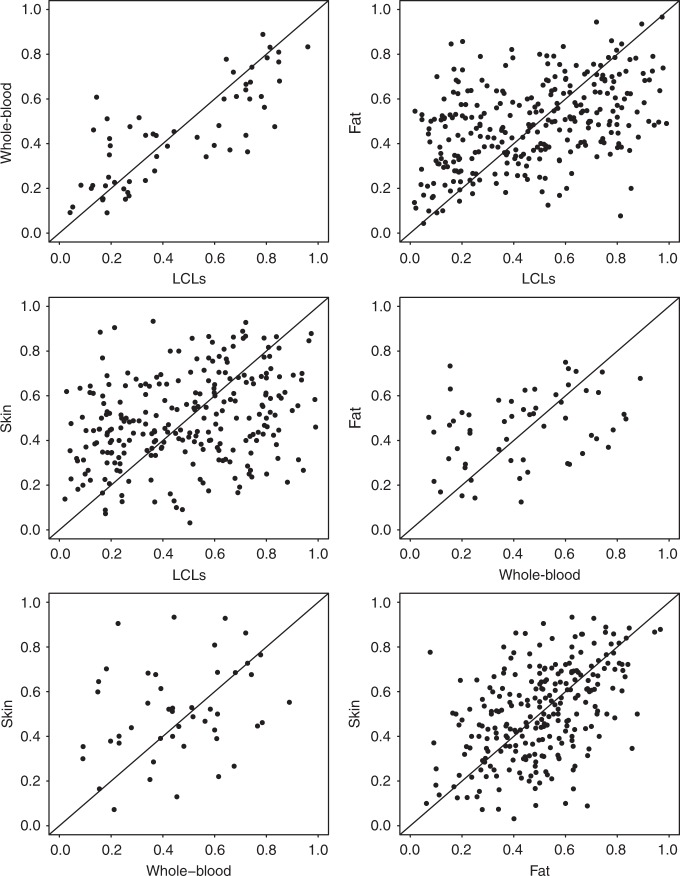
Tissue–tissue comparison of *XIST*_ASE_ reveals the highest similarity of XCI skew between blood tissues. Each plot shows the comparison of *XIST*_ASE_ between two tissues from an individual. Each dot represents an individual. In each comparison all individuals with available *XIST*_ASE_ calls for both tissues were included. Diagonal line indicates perfect concordance in the data (*ρ* = 1).

We observed a wide range of DS values in the four tissues (Fig. 2), with clear differences in the prevalence of skewed individuals between tissues. Blood-derived tissues had the highest incidence of skewed individuals, with skewed XCI observed in 34% of LCLs samples and 28% of whole-blood samples and a lower incidence in the primary tissues, where 12% of fat and 16% of skin samples exhibited skewed XCI (Table 1). In order to examine the extent of similarities of XCI patterns between tissues, we compared the tissue-specific XCI skewing levels in a pairwise manner (Fig. 3). For each tissue–tissue comparison, we included individuals with *XIST*_ASE_ calls in both tissues (Table 2). We found the strongest correlation on *XIST*_ASE_ calls between LCLs and whole blood (*n* = 59, Spearman’s *ρ* = 0.78, *P* = 2 × 10^−13^), indicating that blood-derived tissues share highly similar XCI skewing levels. We also found a good degree of similarity between the XCI skewing levels in fat and skin tissues (*n* = 252, Spearman’s *ρ* = 0.47, *P* = 2 × 10^−15^; Fig. 3). However, low concordance was observed between skin and whole blood (*n* = 47, Spearman’s *ρ* = 0.3, *P* = 0.04) and fat and whole-blood (*n* = 57, Spearman’s *ρ* = 0.33, *P* = 0.01). Our data demonstrate that tissue-specific XCI skewing within an individual is common in the population, indicating that XCI patterns are partially controlled by tissue-specific regulatory mechanisms.

**Table 1:** Prevalence of skewed X-inactivation (XCI) differs across tissues in the TwinsUK cohort.

Tissue	Informative individuals	Skewed XCI individuals	Prevalence of skewed XCI
LCLs	422	145	34%
Whole blood	72	20	28%
Fat	378	45	12%
Skin	336	54	16%

**Table 2:** Correlation of XCI skew varies between tissue pairs, and is highest between blood-derived tissues.

Tissue	LCLs	Whole blood	Fat	Skin
LCLs	1	0.78 [2e−13]	0.42 [5e−14]	0.3 [1e−06]
Whole-blood	0.78 [2e−13]	1	0.33 [1e−02]	0.3 [4e−02]
Fat	0.42 [5e−14]	0.33 [1e−02]	1	0.47 [2e−15]
Skin	0.3 [1e−06]	0.3 [4e−02]	0.47 [2e−15]	1

Coefficient of correlation [and its *p*-value] of XCI skew between tissue pairs. *n*_LCLs-Whole_blood_ = 59; *n*_LCLs-Fat_ = 288; *n*_LCLs-Skin_ = 252; *n*_whole_blood-Skin_ = 47; *n*_whole_blood-Fat_ = 57; *n*_Fat-Skin_ = 252

The active or inactive state of each X chromosome in a cell is clonally passed on to daughter cells. In a pool of cells derived from a single clone (or patch), the XCI patterns are expected to be completely skewed. Patch size refers to the amount of cell clones in a pool of cells (e.g. in a tissue biopsy). We considered the possibility that patch size might bias our quantification of XCI patterns in fat and skin samples. This is likely to occur in biopsies that are smaller than the tissue patch size. However, several considerations led us to exclude the possibility that patch sizes in fat and skin biopsies might confound our *XIST*_ASE_ calls. First, the biopsies included skin samples of 8 mm^3^ in size, which were cut into two skin and three fat samples. As reported in another study, this size is large enough to measure the XCI ratio without being confounded by patch size^42^. Second, most individuals exhibit random XCI patterns in fat and skin tissues, which is unlikely if patch size was larger than the biopsies. We therefore conclude that the biopsies used in this study are large enough to accurately assess the XCI patterns without being biased by patch size.

### LCLs in this study are representative of XCI skewing in-vivo in blood tissues

LCLs generated by Epstein–Barr virus (EBV) mediated transformation of B-lymphocyte cells have been and are widely used in gene expression studies. However, the possibility that the cell lines are monoclonal and/or polyclonal due to selection in the transformation process or clonal expansion in cell culture, and hence not be representative of the in vivo XCI skewing levels, is a potential problem when using LCLs to assess XCI skewing^43^. As the profiled RNA in this study was extracted from the LCLs very shortly after transformation with limited passaging or time in culture we expected this effect to be minimal, however, to address the possibility we performed the following analyses. First, as described above and shown in Fig. 1a, the degree of skewing in LCLs were highly correlated with the HUMARA-based quantifications of XCI patterns in paired whole-blood samples (Spearman’s *ρ* = 0.8, *P* = 7 × 10^−5^, *n* = 18). We would not expect such high similarity between the two quantifications if clonal propagation had occurred in LCLs samples after preparation. This was confirmed by the high correlation between LCLs and whole-blood *XIST*_ASE_ values (Spearman’s *ρ* = 0.78, *n* = 59; Fig. 3) and overall similarity in the prevalence of skewed XCI in LCLs and whole blood (Table 1). Finally, we assessed the degree of skewing in monocytes, B, T-CD4^+^, T-CD8^+^ and natural-killer (NK) cells purified from two monozygotic (MZ) twins exhibiting skewed XCI patterns in LCLs and from one individual exhibiting random XCI patterns in LCLs (Supplementary Fig. 5). We found that in both MZ twins showing skewed XCI in LCLs, the majority of immune cell types exhibited skewed XCI patterns. Conversely, none of the immune cell types purified from the nonskewed individual exhibited skewed XCI patterns (Table 3). These data indicate that within an individual, XCI skewing levels are shared across hematopoietic cells. We conclude that the XCI skewing levels of LCLs in this study are representative of XCI skewing in vivo in blood tissues.

**Table 3:** XCI skew in LCLs in this study is representative of XCI in purified primary immune cells.

Cell types	Degree of skewing in Twin A	Degree of skewing in Twin B	Degree of skewing in Individual C
LCLs	0.45	0.45	0.1
Monocytes	0.43	0.42	0.05
B cells	0.4	0.42	NA
T-CD4^+^ cells	0.28	NA	0.05
T-CD8^+^ cells	0.44	0.3	0.06
NK cells	0.4	NA	0.01

Degree of skewing of XCI in immune cell types purified from two monozygotic twins (Twin A and Twin B) exhibiting skewed XCI patterns in LCLs, and from one individual (Individual C) exhibiting random XCI pattern in LCLs. Degree of skewing ≥ 0.3 indicates skewed XCI patterns

### XCI skewing levels are positively associated with age in blood-derived tissues

XCI skewing levels in peripheral blood have been shown to increase with age in multiple studies^18–22,24,40,44,45^. The age-related increase of XCI skewing levels continues throughout life, since centenarians exhibit higher XCI skewing levels than 95 years old females^22^. However, there is very limited knowledge on the relationship between XCI patterns and ageing in tissues other than blood. In order to explore this, we investigated the association between age and degree of skewing in LCLs, fat, and skin. Our whole-blood estimates were excluded from analysis due to the low sample size (*n* = 72). Age was positively associated with XCI skew in LCLs (*n* = 422, *P* < 0.01), but we did not detect any association between XCI skew and age in skin (*n* = 336, *P* = 0.4) or in fat (*n* = 378, *P* = 0.7).

**Fig. 4:**
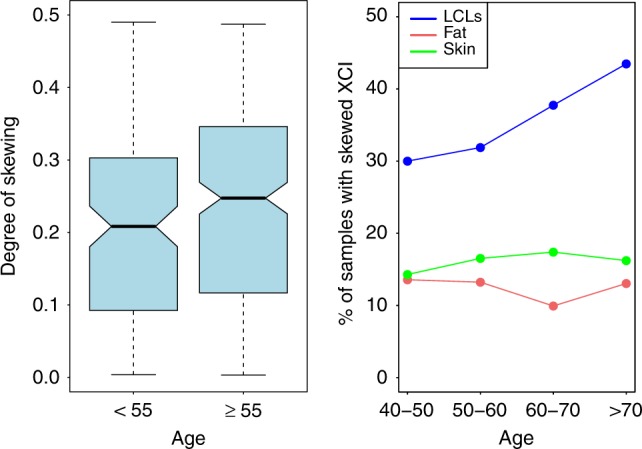
Skewed XCI increases with age in blood-derived tissues but not in fat or skin tissues. Left panel, boxplots show the degree of skewing in LCLs samples <55 (*n* = 141) and ≥ 55 (*n* = 281). Median (black line in the middle), first and third quartile (boxplot bounds), and whiskers (dashed lines) are shown. Right panel, line plots show the frequencies of skewed XCI at different ages in LCLs (blue), fat (red) and skin (green) tissues. XCI X-chromosome inactivation.

We next explored the dynamics of DS and age progression in each tissue, using the lowess procedure. Lowess curve detected an increase of DS beginning at around 55 years old in LCLs (Supplementary Fig. 6), in agreement with what was found in other studies^20,22^. Since the increase of DS starts at around 55 years, we divided LCLs samples into a younger group (*n* = 141, age < 55) and an older group (*n* = 281, age ≥ 55). We found that the mean DS in LCLs was significantly higher in older than in younger females (DS_younger_ = 0.2, DS_older_ = 0.24, *T* test, *P* = 0.03; Fig. 4). Accordingly, we found that the frequency of skewed XCI in LCLs was significantly higher in older (38%) than in younger (28%) females (*χ*^2^ test, *P* = 0.04; Fig. 4). In agreement with the lack of association between the DS and age, we did not detect significant differences between the mean DS in young and older females in fat (DS_younger_ = 0.15, DS_older_ = 0.15) or in skin tissues (DS_younger_ = 0.16, DS_older_ = 0.17). To acquire a more detailed view of the tissue-specific prevalence of skewed XCI in different groups of age, we categorized the samples into four age groups (40–50, 50–60, 60–70, and >70) and calculated the frequency of skewed XCI in each category (Fig. 4). We found that the frequency of skewed XCI increased with age in LCLs, with 41% of individuals >65 years old demonstrating skewed XCI patterns. We did not observe any increase in the skewed XCI frequencies with age in fat and skin tissues. Overall, these data further confirm that XCI skewing levels increase with age in blood-derived tissues, supporting previous investigations. However, we find that there is no increase in XCI in fat and skin tissue from the same individuals, suggesting that acquired XCI skewing with age is a distinctive feature of blood-derived tissues.

### Heritability of skewed XCI is dependent on tissue and age

Twin studies are a powerful strategy to investigate the heritability of complex traits. Previous twin studies have reported that skewed XCI in blood-derived samples is heritable, with *h*^2^ estimates of 0.68 in granulocytes of elderly twin pairs and 0.58 in peripheral blood cells^22–24^, however, these studies have not investigated heritability outside of blood. To estimate the influence of additive genetic effects (heritability) and environmental factors on the observed variance in XCI in the three tissues, we implemented the ACE twin model. The ACE statistical model quantifies the contribution of additive genetic effects (A), shared environment (C) and unique environment (E) to the phenotype variance. In order to investigate whether heritability varies with age, we stratified the twin pairs into a younger group (age < 55) and an older group (age ≥ 55; Supplementary Table 1). Age 55 was chosen as it was identified as the inflection point at which XCI skew begins to increase in the lowess analysis (Supplementary Fig. 6), in agreement with other studies^20,22^. We found that XCI skewing is heritable in LCLs of older females (ACE model, *h*^2^ = 0.34, *P* = 9.6 × 10^−6^), but not younger females (*h*^2^ = 0, *P* = 1). There was no evidence of heritability of XCI skew in fat or in skin tissues at any age (Table 4). The highest proportion of variance was explained by unique environmental factors in all tissues of both younger and older females (*E*^2^_LCLs_younger_ = 0.99, *E*^2^_LCLs_older_ = 0.66, *E*^2^_Fat_younger_ = 0.73, *E*^2^_Fat_older_ = 0.92, *E*^2^_Skin_younger_ = 1, *E*^2^_Skin_older_ = 1). As a complement to the heritability analysis, we calculated the intraclass Spearman correlation (IC) of XCI skew within MZ and DZ twin pairs of all ages, and within younger and older MZ and DZ twin pairs (Table 5). IC analyses of twin pairs is often used to demonstrate the existence of genetic effect in smaller sample sizes. The IC of XCI skew within MZ twins pairs was positive and statistically significant (IC_MZ_allAges_ = 0.31, *P* = 0.02). We found significant IC of XCI skew within older MZ twin pairs (IC_MZ_older_ = 0.42, *P* = 0.005), but not within young MZ twin pairs (IC_MZ_younger_ = 0.06, *P* = 0.8). We did not detect significant IC within DZ twin pairs at any age, in agreement with previous study in blood^22^. The higher IC of XCI skew within MZ twin pairs compared with DZ twin pairs indicates the involvement of genetic determinants in the regulation of XCI skew in blood-derived tissues. The increase of IC in older compared to younger MZ twin pairs and the fact that the heritability of XCI skew is observed only in females older than 55, confirm a role for genetic variants as age-dependent regulators of the acquired XCI skew in blood-derived tissues. Presumably, genetically-determined secondary cell selection processes act in haematopoietic cell lineages, with the high mitotic rates contributing to the manifestation of their effects in blood-derived tissues. Results also highlight an age-independent role for environmental factors as regulators of XCI skew in blood, fat, and skin tissues.

**Table 4:** XCI skew is heritable in blood-derived tissues of older females.

Tissue [age group]	Additive genetics (*h*^2^)	Common environment	Unique environment
LCLs [<55]	0	0.01	0.99
LCLs [≥55]	0.34	0	0.66
Fat [<55]	0	0.27	0.73
Fat [≥55]	0	0.08	0.92
Skin [<55]	0	0	1
Skin [≥55]	0	0	1

Estimates of the relative contribution of additive genetics, common environment and unique environmental factors to the tissue-specific XCI skew in age-stratified twins

**Table 5:** Intraclass Spearman’s correlations of XCI skew in blood-derived tissues in age stratified twin pairs.

	MZ_allAges_	MZ_young_	MZ_older_	DZ_allAges_	DZ_young_	DZ_older_
n. pairs	61	18	43	63	25	38
Intraclass correlation	0.31	0.06	0.42	0	0.1	−0.1
*P*-value	0.02	0.8	0.005	0.9	0.6	0.7

Twin pairs < 55 years old are classified as young, twin pairs ≥ 55 years old are classified as old

### Individuals with autoimmunity exhibit more skewed XCI than unaffected co-twins

Chomosome X is enriched for immune-related genes. Most autoimmune disorders have higher prevalence in females than males^17^. Klinefelter syndrome (47,XXY) males have up to 14-fold higher risk of autoimmunity than 46,XY males^46,47^. These observations support an X-dosage effect in the pathogenesis of autoimmune diseases. As a mechanism of X-dosage compensation, the XCI process could be involved in the etiology of autoimmune disorders. Unbalanced X-linked transcriptional dosages toward one parental haplotype caused by skewed XCI patterns could influence the functions of the immune system. In particular during development, random XCI patterns in dendritic cells allow balanced expression of both parental X-linked self-antigens, a crucial event for the identification and negative selection of autoreactive T-cells in the thymus^16,17^. In line with the loss of mosaicism hypothesis^48^, we postulated that skewed XCI patterns may promote a breakdown of the tolerance induction processes with consequent release of autoreactive immune cells into the circulatory system. Supporting this hypothesis, higher frequencies of skewed XCI patterns have been observed in females affected with autoimmune disorders than in healthy controls^12,13^, however it is not known if this is a cause or consequence of disease, and these studies have not taken into account the underlying genetic predisposition of the cases and controls.

**Fig. 5:**
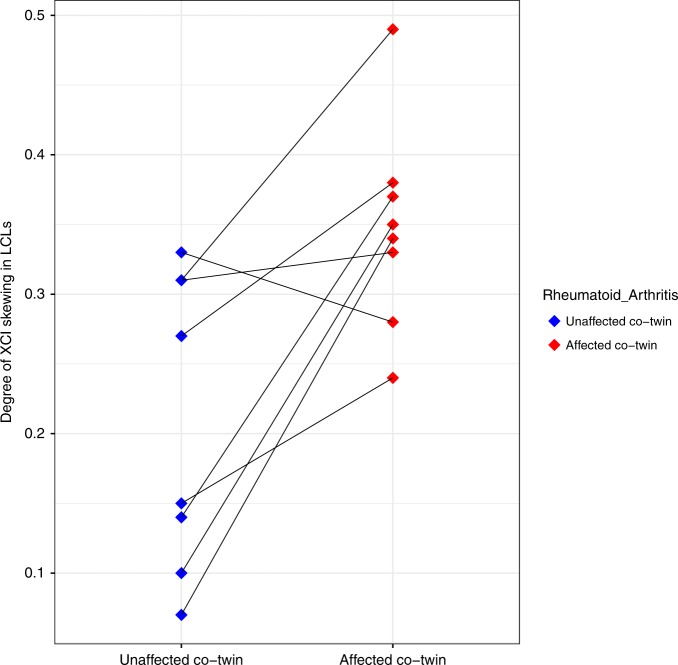
XCI patterns are more skewed in blood-derived tissues of individuals affected with rheumatoid arthritis than in their unaffected identical co-twins. *Dotplot* shows the degree of XCI skewing in LCLs from monozygotic twin pairs that are discordant for rheumatoid arthritis (*n* = 8 twin pairs). Blue dots on the left represent the twin without rheumatoid arthritis and red dots the co-twins diagnosed with rheumatoid arthritis. Co-twins are connected by a line. XCI X-chromosome inactivation.

In order to address the association between autoimmune disease and XCI skewing while controlling for genetics, we investigated our samples for MZ twin pairs discordant for autoimmune disease. We identified eight MZ twins pairs discordant for RA in our study. RA is a chronic autoimmune condition affecting the lining of the synovial joints and associated with progressive disability^49^. RA is up to three times more frequent in females than males, and the most common age of onset ranges between 50 and 60^50^. Prevalence of RA ranges from 0.5% to 1%, but it significantly rises with age^51^. We found that the degree of XCI skewing in LCLs significantly differed between unaffected and affected co-twins (mean_DS_healthy_ = 0.21; mean_DS_affected_ = 0.35; paired Wilcoxon’s test *P* < 0.05; Fig. 5). In seven out of eight twin pairs the affected co-twin was more skewed than their unaffected sister. These results are consistent with patterns seen in twins discordant for systemic lupus erythematosus and autoimmune thyroid disease^52,53^. Only four of the eight RA-discordant twin pairs had available XCI skew calls in fat and skin, and there was no significant difference in the XCI skew between the unaffected and affected co-twins in either tissue. Identical twins share 99% of the genome, age, and multiple environmental traits including in-utero growth, early life, and in most cases, socioeconomic statuses. Differences in the XCI patterns in blood may generate differences in the X-linked transcriptional dosages of immune-related genes and affect identical twins differently. Discordance in RA between identical twins could be well explained by differences in XCI patterns, however, we note that with cross-sectional data it is not possible to determine if changes in XCI drive RA or if RA causes changes in XCI skewing, or if both are independently associated to a third factor. Due to the low sample size, we acknowledge these results are not generalizable at a population level; larger sample sizes will be required to fully test the association of skewed XCI with twin discordance for RA and other autoimmune traits.

### XCI skew is associated with smoking status in older females

Tobacco smoking has been reported to induce epigenomic changes including DNA methylation variation (reviewed in ref. ^54^). Smoking is a well characterized risk factor in cancer^55^ and, as more recently discovered, in the etiology of autoimmunity^56^. Although smoking-related X-linked DNA methylation sites have been discovered^57^, no previous studies, to our knowledge, have investigated the relationship between smoking and XCI patterns. We reasoned that changes of XCI patterns may result from smoking, and affect in turn short-term and long-term health. In order to test our hypothesis, we used the 270 individuals in our dataset for which we had smoking status at the time of sample collection, including 233 never smokers and 37 current smokers^58^. We found no difference in the frequency of skewed XCI patterns between never and current smokers (36% and 35%, respectively) in LCLs. To take into account the effects of age on the degree of skewing in blood-derived tissues and to examine the relationship between smoking status and degree of skewing at different ages, we split the dataset into a younger (age < 55) and an older group (age ≥ 55; Supplementary Table 2). While the frequencies of skewed XCI were very similar between young smokers and young never smokers (27% and 28%, respectively), we detected a higher prevalence of skewed XCI in older smokers compared with older never smokers (47% and 40%, respectively). Accordingly, we found an overall positive association between XCI skew and smoking status in older (*P* = 0.02), but not in younger individuals (*P* = 0.5). The data suggest a role for smoking as a modulator of XCI skew in blood-derived tissues of females older than 55. Presumably, the association between smoking and XCI skew changes is complex, and further investigations are needed to characterize the genetic and molecular mechanisms underlying this phenomenon.

## Discussion

In this study, we used multi-tissue transcriptomic data from twins to comprehensively characterize XCI patterns in LCLs, whole-blood, fat, and skin tissues from a healthy twin cohort. We show XCI patterns to be tissue-specific and that blood-derived tissues exhibited the highest prevalence of skewed XCI and share the highest similarity of XCI patterns. These findings indicate that XCI patterns are partially driven by tissue-specific mechanisms, and that the XCI skew measured in blood is not a reliable proxy for the skew in other tissues. Skewed XCI patterns limited to disease-relevant tissues and cells have been observed in multiple conditions^9,10,14,15,59,60^ but except for several cases of X-linked diseases, their roles in disease etiology and predisposition remain largely unknown. Our results demonstrate that tissue-specific XCI patterns within an individual is common in this healthy population.

We show that XCI skewing levels in blood tissues increase with age, with an inflection point at around 55, in line with previous reports^18–22,24,40,44,45^. In this study, more than 41% of females >65 years old demonstrate skewed XCI patterns in blood-derived tissues, indicating that acquired skewed XCI is a highly prevalent phenotype in ageing populations. We show age-related increase in XCI skew is a distinctive feature of blood-derived tissues, with no evidence for an age-related increase in fat or skin. Age-related increase in XCI skew partially explains the higher incidence of skewed XCI in blood than fat and skin tissues. The effects of age-related skewing of XCI on healthy ageing remain largely unknown, but may have a broad impact on the immune system. We demonstrate that within an individual, the XCI patterns in blood-derived tissues are shared across multiple immune cell types including monocytes, B-cells, T-CD4^+^, T-CD8^+^ and NK cells. Hematopoietic stem cells and the immune system continue to develop throughout life. Presumably, in line with the loss of mosaicism hypothesis^48^, imbalanced X-linked immune-related gene expression toward one parental haplotype leads to a reduced molecular diversity, which may translate in a decline of immune repertoire as well as poor sustenance of the immunological memory. Thus, by influencing the immune system, age-acquired skewed XCI could influence the predisposition to and manifestation of age-related traits, such as hematopoietic disorders, in women. We support an involvement of skewed XCI in the regulation of the immune system by showing that XCI patterns in LCLs, and consequently in multiple immune cell types, are consistently more skewed in individuals affected with autoimmunity than in healthy identical co-twins. Identical twins share nearly 100% of the genome, including chromosome X. Discordance in an autoimmune phenotype between twins, could partially be attributed to differences in the X-linked allelic and transcriptional dosages of X-linked immune-related genes resulting from difference in the XCI skew.

Previous twin studies have reported that XCI patterns in blood have a genetic component^22,24^. To our knowledge, this is the first study to investigate heritability of XCI skewing levels in other tissues. We found that the heritability of XCI skewing level is limited to blood-derived tissues of females >55 years old (*h*^2^ = 0.34), with no evidence of heritability in fat or skin or younger individuals in any tissue. The restriction of heritability to blood of older individuals is of interest given the link between skewed X-inactivation and clonal haematopoiesis. Positive selection of cells carrying an advantageous somatic mutation will lead to clonal haematopoiesis and skewed XCI patterns as the selected cells will carry the same inactivated parental X. Somatic mutation-driven clonal haematopoiesis is now known to be common in blood of healthy older individuals and is often referred to as clonal haematopoiesis of indeterminate potential (CHIP)^36,61–63^. CHIP is associated with increased risk of both cancer and all-cause mortality, and likewise skewed XCI, the prevalence of CHIP increase with age^64,65^. The higher skew in the XCI patterns in individuals affected with RA than in unaffected co-twins, is well explained by both the occurrence and the age-related increase in the prevalence of clonal hematopoiesis in RA patients^66^. The increase in XCI skew in older smokers in our study is also consistent with the increase in clonal haematopoiesis observed in smokers^63,67,68^. All together, these data converge in suggesting a link between XCI skew and clonal hematopoiesis. It is currently unknown to what extent CHIP accounts for age-acquired XCI skew, however, if it is a major driver this would suggest that like age-related XCI skew, CHIP has a significant germline genetic component. Stochastic selection of cells could also contribute to the variance of XCI skewing levels, but, in agreement with previous works^22,24^, we reason that their contribution is minimal. If stochastic selection of cells was a dominant mechanism, the correlation of XCI patterns between twin pairs would decrease with age.

Overall, the data presented in this study indicate a gene × age interaction that shifts the functional allelic dosages of X-linked heterozygous loci in a tissue-restricted manner. The high prevalence of skewed XCI and tissue-restricted XCI in a healthy population could complicate discovery of Chromosome X variants associated with a trait and subsequent genetic risk prediction, as an individual’s genotype may not match their functional genotypic dosage in the relevant tissue. Further investigations of the heterogeneity of XCI patterns across tissues and how this is regulated are essential to clarify the biomedical implications of skewed XCI and its role in healthy ageing in women.

## Methods

### Sample collection

The study included 856 female twins from the TwinsUK registry^29,30^ who participated in the MuTHER study^69^ . Study participants included both MZ and dizygotic (DZ) twins, aged 38–85 years old (median age = 60; Supplementary Fig. 1) and were of European ancestry. Volunteers received detailed information regarding all aspects of the research project and gave a prior signed consent to participate in the study. Peripheral blood samples were collected and LCLs were generated via EBV-mediated transformation of the B-lymphocyte fraction. Punch biopsies of subcutaneous adipose tissue were taken from a photo-protected area adjacent and inferior to the umbilicus. Skin samples were obtained by dissection from the punch biopsies. Adipose and skin samples were weighed and frozen in liquid nitrogen. This project was approved by the research ethics committee at St Thomasʼ Hospital London, where all the TwinsUK biopsies were carried out. Volunteers gave informed consent and signed an approved consent form prior to the biopsy procedure. Volunteers were supplied with an appropriate detailed information sheet regarding the research project and biopsy procedure by post prior to attending for the biopsy.

### Genotyping and phasing

Whole-Genome Sequence data were generated and genotypes called within the UK10K project^70^. Briefly, DNA was sheared to 100–1000 bp and used for a paired-end library preparation. DNA libraries were sequenced on a Illumina HiSeq machine and 100 bp paired-end reads were generated. Reads were aligned to the GRCh37 reference genome using the Burrows-Wheeler Aligner^71^. SAM to BAM format conversion and sorting were performed using either Picard (broadinstitute.github.io/picard/) or samtools^72^. Variants were called from pooled alignments with samtools and bcftools (www.htslib.org/doc/bcftools.html) and VCF files were generated. For chrX and chrY variant calls, PAR of chrY was masked and male samples were called as diploid in their X-linked PAR and haploid in non-PAR regions. Sites were recalled and recalibrated with GATK UnifiedGenotyper and VariantRecalibrator^73^. SNV with a VQSLOD (variant quality score odds ratio) corresponding to a truth sensitivity <99.5% and with a Hardy–Weinberg equilibrium *P*-value < 1e−6 were discarded. Overall sequencing accuracy was assessed by comparing genotype concordance on chromosome 20 in 22 MZ twin pairs. Five hundred and fifty-seven individuals had both X-chromosome sequence data and RNA-seq data for at least one tissue. For individuals with unavailable X-chromosome sequence data, X-linked genotypes data were retrieved from the TwinsUK genotypes previously imputed into the 1000 Genomes Project phase 1 reference panel^74–76^. Haplotypes of X-linked SNPs with a MAF >5% were then phased using shapeit v2.r837^77,78^, with the–chrX flag to set up all functionalities for the phasing of non-PARs of the X chromosome, along with a phasing window of 2 Mb and 1000 conditional states. Phasing was performed using the genetic map b37 and the 1000 Genomes Project phase three reference panel of non-PAR X-linked haplotypes^78^. Phased X-linked SNPs were used for further analysis.

### RNA-sequencing data

The Illumina TruSeq sample preparation protocol was used to generate the cDNA libraries for sequencing. Samples were sequenced on an Illumina HiSeq 2000 machine and 49 bp paired-end reads were generated. Adapter and polyA/T nucleotide sequences were removed and sequencing reads were aligned to the UCSC GRCh37/hg19 reference genome with the Burrows-Wheeler Aligner v.0.5.9^71^. Samples that failed library preparation (according to the manufacturer’s guidelines) or had less than 10 million reads were discarded. Genes were annotated using the GENCODE v10 reference panel^79^.

### Longitudinal RNA-sequencing data

Peripheral blood samples were collected 1–2.7 years apart from 114 female twins of the TwinsUK registry and were processed with the Illumina TruSeq protocol, sequenced on a HiSeq 2000 machine and 49 bp paired-end reads generated^41^. Adapter and polyA/T nucleotide sequences were trimmed using trim_galore and PrinSeq tools^80^, respectively. Reads were aligned to the UCSC GRCh37/hg19 reference genome with the STAR v.2.5.2a aligner^81^. Alignments containing non-canonical and unannotated splice junctions were discarded. Properly paired and uniquely mapped reads with a MAPQ of 255 were retained for further analysis.

### Purified immune cells RNA-sequencing data

Monocytes, B, T-CD4^+^, T-CD8^+^ and NK cells were purified using fluorescence activated cell sorting (FACS) from two MZ twins exhibiting skewed XCI patterns in LCLs and from one individual exhibiting random XCI patterns (Supplementary Fig. 5). Total RNA was isolated and cDNA libraries for sequencing were generated using the Sureselect sample preparation protocol. Samples were then sequenced in triplicates on an Illumina HiSeq machine and 126 bp paired-end reads were generated. Adapter and polyA/T nucleotide sequences were trimmed using trim_galore and PrinSeq tools^80^, respectively. Human and prokaryotic rRNAs were identified using sortmerna v.2.1^82^ and removed. Reads were aligned to the UCSC GRCh37/hg19 reference genome using STAR v.2.5.2a^81^. Alignments containing noncanonical and unannotated splice junctions were discarded. Properly paired and uniquely mapped reads with a MAPQ of 255 were retained for further analysis.

### Correction of RNA-seq mapping biases

To eliminate mapping biases, all RNA-seq data were re-aligned within the WASP pipeline for mappability filtering^83^. The WASP tool has an algorithm specifically designed to identify and correct mapping biases in RNA-seq data. In each read overlapping a heterozygous SNP, the allele is flipped to the SNP’s other allele (generating all possible allelic combinations) and the read is remapped. Reads that did not remap to the same genomic location indicate mapping bias and were discarded. Reads overlapping insertions and deletions were also discarded. Properly paired and uniquely mapped reads were retained for analysis.

### Quantification of allelic read counts and allele-specific expression (ASE)

Allelic read counts at heterozygous SNPs within *XIST* were quantified from paired RNA-seq and X-linked genotypes data using GATK ASEReadCounter^84^. Reads flagged by ASEReadCounter as having low base quality were discarded. No retained reads overlapped more than one heterozygous position in an individual. X-linked SNPs with a read depth of least ten reads were used. To increase the confidence that genotypes were truly heterozygous, only SNPs with both alleles detected in RNA-seq data at least once were retained for analysis, as previously done^30^. Samples with at least 1 heterozygous *XIST*-linked SNP passing all filters were retained as informative of XCI skewing levels (422 LCLs, 72 whole-blood, 378 fat and 336 skin samples). To quantify the ASE of each heterozygous *XIST*-linked SNP, the read count at the reference allele was divided by the read depth at the site^84^. The SNP’s ASE values range from 0 to 1, where 0 and 1 indicate monoallelic expression and 0.5 indicates biallelic expression.

### Quantification of *XIST*_ASE_ and degree of XCI skewing (DS)

In each sample, the XCI skewing levels were quantified by averaging the ASE values of heterozygous SNPs within *XIST*. All SNPs were phased prior to averaging as detailed above. The measure, called *XIST*_ASE_ is defined as follow:

1 $$XIST_{{\mathrm{ASE}}} = \frac{{{\sum} {XIST\_{\mathrm{SNP}}_{{\mathrm{ASE}}}} }}{{n}}\;\left( {XIST_{{\mathrm{ASE}}}} \right),$$

where *XIST*_SNP_ASE_ are the ASE values of heterozygous SNPs within *XIST* and n is the number of heterozygous SNPs within *XIST* in the sample. *XIST* is uniquely expressed from the inactive X chromosome^3,28,31^, and thus the relative expression of parental alleles within *XIST* transcript are representative of XCI skewing levels in a bulk sample. The expression levels of polymorphisms within *XIST* have been used to infer XCI skewing levels in a sample^32,33^. We have also calculated XCI skew using the ASE of all non-PAR genes available in a sample, as previously done^34^. We compared our *XIST*_ASE_ calls to the non-PAR-based calls and concluded that the *XIST*_ASE_ calls are a better proxy for XCI skew in our analyses (Supplementary Note 1, Supplementary Figs. 2–4). Within each sample, the *XIST*_ASE_ values range from 0 to 1; an *XIST*_ASE_ value of 0.5 indicates equal inactivation of the two parental chromosomes (completely random XCI patterns, 50:50 XCI ratio), whereas a value of 0 or 1 indicates complete inactivation of one parental chromosome (completely skewed XCI patterns, 100:0 XCI ratio). To be consistent with previous literature^22,38^, we classified samples with *XIST*_ASE_ ≤ 0.2 or *XIST*_ASE_ ≥ 0.8 to have skewed XCI patterns, and samples with 0.2 < *XIST*_ASE_ < 0.8 to have random XCI patterns. To have an absolute measure of the magnitude of the XCI skewing levels in each sample, (or effect size of *XIST*_ASE_), the degree of skewing of XCI (DS) was calculated. DS is the absolute deviation of *XIST*_ASE_ from 0.5. In each sample, DS was calculated as follow:

2 $${\mathrm{DS}} = \left| {0.5 - XIST_{{\mathrm{ASE}}}} \right|\;\left( {{\mathrm{Degree}}\;{\mathrm{of}}\;{\mathrm{skewing}}\;{\mathrm{of}}\;{\mathrm{XCI}}} \right).$$

DS does not take into account the direction of XCI skewing, but the degree of deviation from a 50% XCI patterns (*XIST*_ASE_ = 0.5). DS is then a measure of the magnitude of XCI skewing levels in a sample. DS values range from 0 to 0.5, where 0 means random XCI and 0.5 completely skewed XCI patterns. Samples with DS ≥ 0.3 were classified to have skewed XCI, while samples with DS <0.3 were classified to have random XCI.

### Heritability analysis of DS

The relative contributions of additive genetic factors (A), shared (C) and unique environmental factors (E) to the tissue-specific variance of DS, were calculated using the *twinlm()* function in the *mets* R package^85^. For each tissue, samples were split into a young (<55) and an older (≥55) group according to their ages (Supplementary Table 1). Due to the low number of MZ and DZ twin pairs in each group, whole-blood was excluded from heritability analysis. To further assess the contribution of genetic effects, the intraclass Spearman’s correlation (IC) of DS in blood-derived tissues of young and older MZ and DZ twin pairs was also calculated.

### Differences in the DS between identical co-twins discordant for RA

We used a subset of eight MZ twin pairs where the co-twins of each pair are discordant for RA. Diagnoses were either confirmed during visits at the rheumatologist clinics at St Thomas’ Hospital in London, or confirmed by phone-interview by a rheumatology clinical fellow to confirm the diagnosis of RA based on the American College of Rheumatology 1987 criteria^86^. In case of unclear diagnosis of RA, participants were reviewed in clinic or were excluded. Difference in the distribution of the degree of XCI skewing in LCLs between the two groups (twins affected with autoimmunity vs healthy co-twins) was evaluated using paired Wilcoxon test. A P-value ≤ 0.05 was considered to be statistically significant.

### Association between degree of skewing and smoking status

Association between the degree of skewing in LCLs and self-reported smoking status was tested in the 270 individuals with reliable smoking status recorded^58^. Dataset included 270 females classified either as current smokers (*n* = 37) or never smokers (*n* = 233; Supplementary Table 2). To examine the association between DS and smoking status, the smoking status was converted into a binary trait (0 = no smoker, 1 = smoker). A linear model of the DS as a function of the smoking status was then implemented for younger (age < 55) and older (age ≥ 55) individuals separately. Age was used as covariate. A *P*-value ≤ 0.05 was considered to be statistically significant.

### Reporting summary

Further information on research design is available in the Nature Research Reporting Summary linked to this article.

## Supplementary information

Supplementary Information

Reporting Summary

## Data Availability

TwinsUK RNA-seq data is available from EGA (Accession number: EGAS00001000805). TwinsUK genotypes and phenotypes are available upon application to TwinsUK Data Access Committee (https://twinsuk.ac.uk/resources-for-researchers/access-our-data/). All other data are contained in the manuscript and its supplementary information.
