# Supplementary material for: Heritability of skewed X-inactivation in female twins is tissue-specific and associated with age
Source: Nat Commun. 2019 Nov 25;10:5339. doi: 10.1038/s41467-019-13340-w (PMC6877649; doi:10.1038/s41467-019-13340-w)
Supplement: Supplementary file 1 — Supplementary Information [file 41467_2019_13340_MOESM1_ESM.pdf]

Heritability of skewed X-inactivation in female twins is tissue-specific and associated with age

Antonino Zito[1], Matthew N. Davies[2], Pei-Chien Tsai[1,3,4], Susanna Roberts[5], Rosa Andres-Ejarque[6], Stefano Nardone[7], Jordana T. Bell[1], Chloe C. Y. Wong[5] & Kerrin S. Small[1].

[1] Department of Twin Research & Genetic Epidemiology, King's College London, London SE1 7EH, UK
[2] Ervaxx Limited, 71 Kingsway, London WC2B 6ST, UK
[3] Department of Biomedical Sciences, Chang Gung University, Taoyuan, Taiwan
[4] Genomic Medicine Research Core Laboratory, Chang Gung Memorial Hospital, Linkou, Taiwan
[5] Institute of Psychiatry, Psychology & Neuroscience, King's College London, London SE5 8AF, UK
[6] St John's Institute of Dermatology, Faculty of Life Science & Medicine, King's College London, London SE1 9RT, UK
[7] Division of Endocrinology, Diabetes, and Metabolism, Department of Medicine, Beth Israel Deaconess Medical Center, Harvard Medical School, Boston, MA 02215, USA

Supplementary Note 1

The rationale of using *XIST*$_{ASE}$ from bulk RNA-seq as proxy for XCI-skew in a sample

Several recent papers[1,2] have used RNAseq and ASE data to infer XCI-skewing, but there is no consensus on how to best apply this methodology. The two most common approaches are the 1) use a monoallelically expressed chrX gene (as we have done using *XIST*) and 2) to use all nonPAR loci available in a sample. The former has the advantage that it is focused solely on a gene that has extensive evidence of complete mono-allelic expression in a given cell, but can be limited in the number of informative individuals as it requires heterozygous expressed sites within *XIST*. The second approach can estimate skew in a larger number of individuals but can potentially underestimate or bias XCI-skew if the additional X-linked SNPs lie in genes that escape X-inactivation. The full extent of XCI escape across tissues and environments is not known.

A bulk tissue is a mosaic of cells with either parental X silenced. Consequently, the allele-specific expression (ASE) of monoallelically expressed X-linked heterozygous sites reflects the XCI-skew in the sample. Conversely, the ASE of genes who are not fully subject to XCI and thus exhibit biallelic expression, may not reflect the XCI patterns in a bulk sample. Biallelic expression is widespread across the X, with over 15% of genes escaping XCI. Genes biallelically expressed are located in both PAR regions and nonPAR regions, with Xi expression ranging from 10% to 80% Xa expression[1]. Thus, the ASE of nonPAR X-genes may not accurately reflects the XCI-skew in a sample as likely biased by biallelically expressed nonPAR X-genes. The rationale of using the ASE of heterozygous SNPs within *XIST* relies on the evidence that *XIST* is exclusively transcribed from the inactive X chromosome (Xi), and thus monoallelically expressed within a cell. The GTEX study[1] confirmed this unique *XIST* behaviour in both bulk RNAseq and single-cell RNAseq. *XIST* is essential for XCI; it binds only in cis on Xi without touching or being expressed from the other X within the cell nucleus[3,4]. It has experimentally been proven that skewed XCI patterns can be detected and quantified solely by assessing the expression levels of XIST-linked heterozygous variants[5]. Furthermore, several studies have supported the HUMARA calls (which represent an indirect measure of XCI patterns), with transcriptional assays based on single monoallelically expressed genes, like *XIST*[6,7]. Using the same gene (*XIST*) in all samples produces XCI-skew calls that are directly comparable between samples. Conversely, when using all nonPAR genes available in a sample, the XCI-skew calls will likely be based on different genes in different samples (as the genes with informative heterozygous SNPs will vary between individuals), which may lead to inconsistency when comparing across individuals.

To compare the two methods, *XIST* only vs nonPAR genes, we regenerated the XCI-skew calls in all samples using the nonPAR method used in Shvetsova, et al[2]., median ASE of all nonPAR heterozygous SNPs demonstrating RNAseq read depth ≥10 reads and with both alleles observed at least once. We used these nonPAR XCI-skew calls to perform the following analyses:

1) evaluate concordance with HUMARA-based XCI-skew calls;
2) assessment of concordance in the XCI-skew at two close time points;
3) comparison with *XIST*$_{ASE}$ –based XCI-skew calls in all tissues.

Compared with *XIST*$_{ASE}$-based XCI-skew calls the inclusion of additional (nonPAR) loci in the calculation of XCI-skew reduces both the degree of replicability with HUMARA from 0.8 to 0.71 (Supplementary Figure 2), and the concordance at two close time points decreased from 0.94 to 0.66 (Supplementary Figure 3). Comparing the *XIST*$_{ASE}$ to the nonPAR XCI skew, we demonstrate that: 1) *XIST*$_{ASE}$-based XCI-skew calls cover a wider range of values; 2) nonPAR$_{ASE}$ reduces the prevalence of skewed XCI in all 4 tissues (Supplementary Figure 4).

With these benchmarking analyses, we demonstrate that the ASE of heterozygous SNPs within *XIST* is a more accurate proxy for the XCI-skew than nonPAR ASE in our dataset. The decrease in accuracy of the nonPAR calls could be due to biological or technical factors, or a combination therein. Biologically, nonPAR genes which escape X-inactivation will bias the estimate of XCI-skew. Technically, we note that our samples are phased with SHAPEIT2, which is the gold standard phasing algorithm for population level phasing. However, while SHAPEIT2 is very accurate across the length of an entire chromosome haplotype switch errors are nearly certain to occur. For reference, in simulated datasets the SHAPEIT2 authors found that ~4 switch errors will occur on chromosome 10 (which is 133 MB, compared to ChrX 155 MB)[8]. A undetected haplotype switch error would switch the reference ChrX in the ASE calls, and thereby confound the ability to estimate XCI-skew and lead to underestimation of XCI-skew. Haplotype switch errors are not an issue in datasets where phase was defined by parental transmission, such as the Shvetsova paper[2] where the genotype of both parents was available.

Supplementary Figures

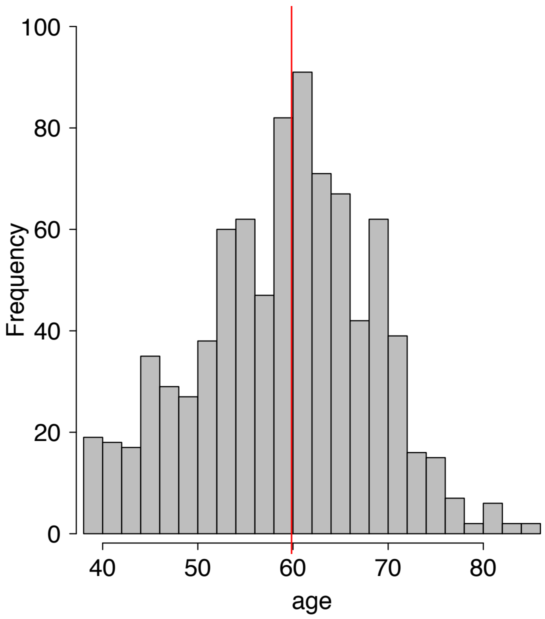

Supplementary Figure 1. Distribution of ages in the TwinsUK samples used in this study. Red line represents the median age.

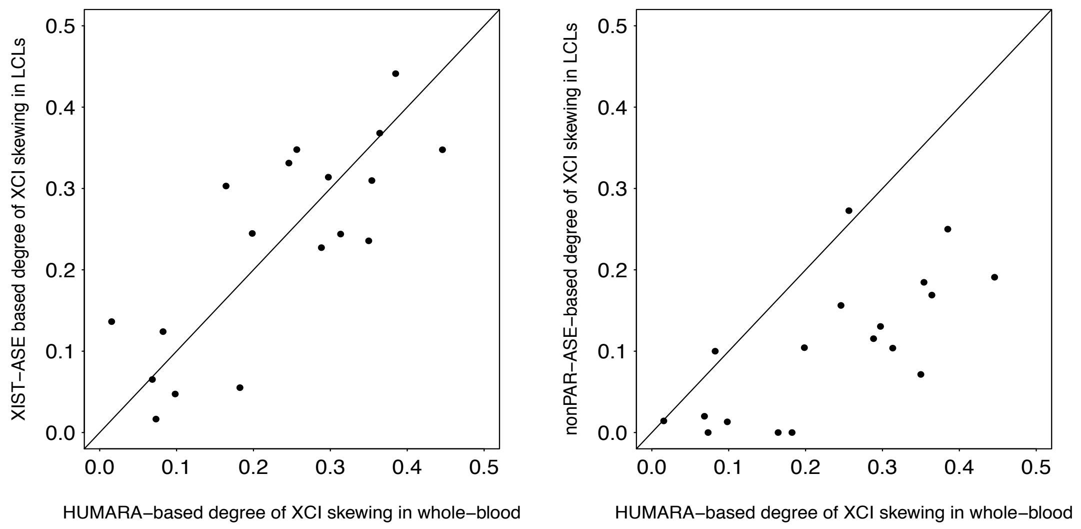

Supplementary Figure 2. Scatter plot of the XCI-skew in 18 LCLs and whole-blood samples quantified via the 2 methods ($XIST_{ASE}$ and $nonPAR_{ASE}$) and HUMARA, respectively. Diagonal line indicates perfect concordance in the data (ρ =1). Including nonPAR SNPs reduces the correlation from 0.8 to 0.71. The decrease in correlation is largely driven by nonPAR underestimating skew compared to HUMARA.

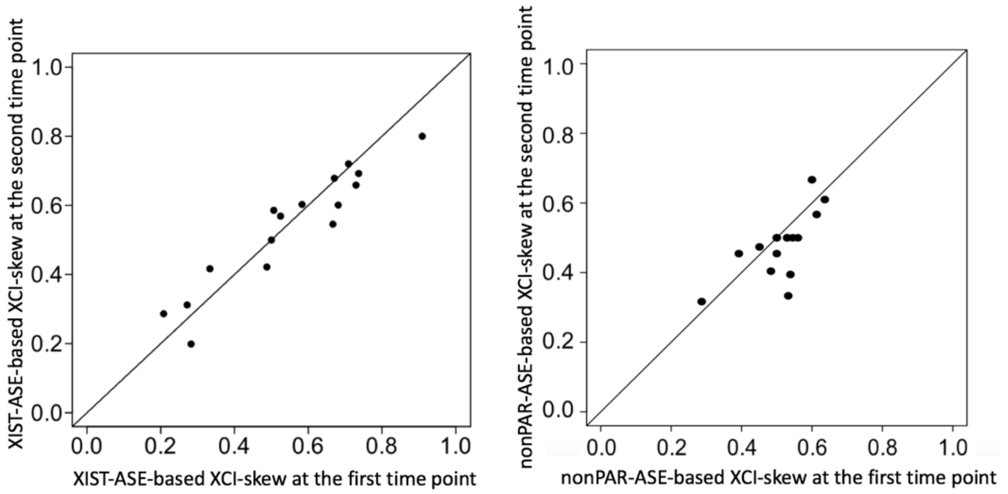

Supplementary Figure 3. Scatter plot of the XCI-skew calls at time point 1 and time point 2 in 16 whole-blood samples quantified using $XIST_{ASE}$ and $nonPAR_{ASE}$. Diagonal line indicates perfect concordance in the data. (ρ=1). Including nonPAR SNPs reduces the correlation from 0.94 to 0.66.

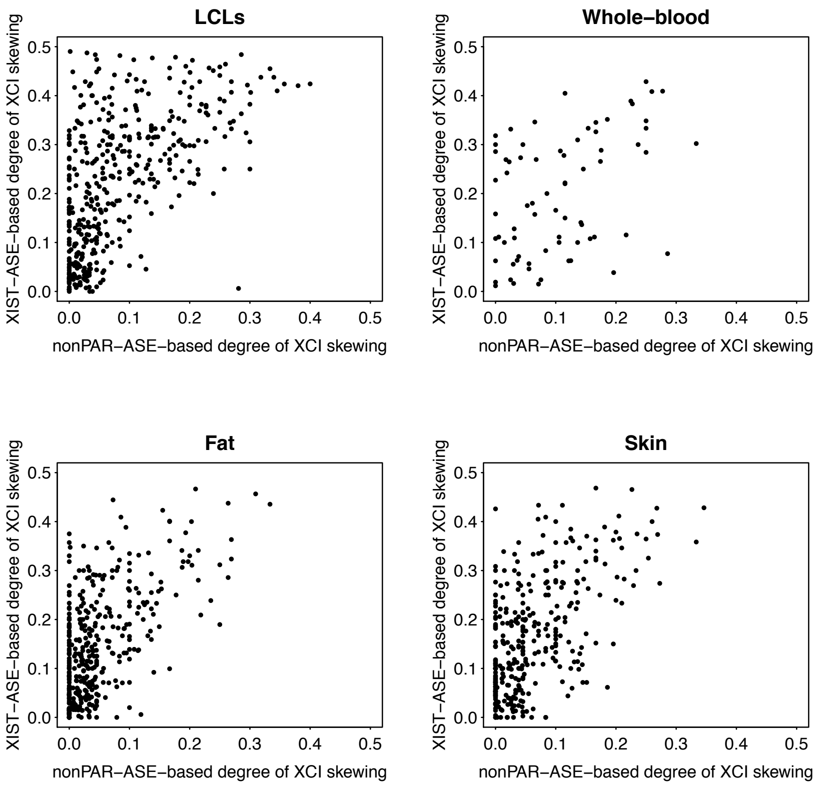

Supplementary Figure 4. Scatter plot of the XCI-skew calls generated via the two methods (*XIST*$_{ASE}$ and nonPAR$_{ASE}$) across all tissue samples (Spearman's ρ_{LCLs}=0.6; $\rho_{whole\text{-}blood}$=0.42; ρ_{Fat}=0.5; ρ_{Skin}=0.5). In each plot, each dot represents an individual. Degree of skewing values ≥0.3 indicate skewed XCI patterns (XCI ratio ≥80:20).

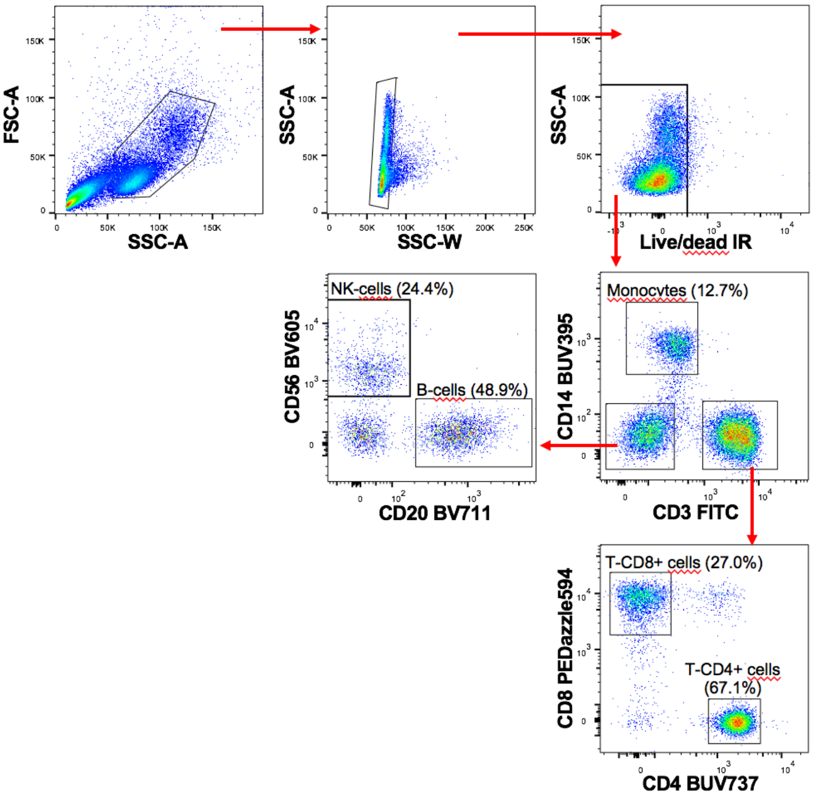

Supplementary Figure 5. Gating strategy used for cell sorting. Gating strategy used to sort monocytes (CD14+), B-cells (CD14-, CD3-, CD56-, CD20+), NK-cells (CD14-, CD3-, CD20-, CD56+), T-CD4+ cells (CD14-, CD3+, CD8-, CD4+) and T-CD8+ cells (CD14-, CD3+, CD4-, CD8+) from freshly isolated PBMCs from 2 monozygotic twins exhibiting skewed XCI patterns in LCLs and 1 individual exhibiting random XCI patterns in LCLs (Table 3 in manuscript).

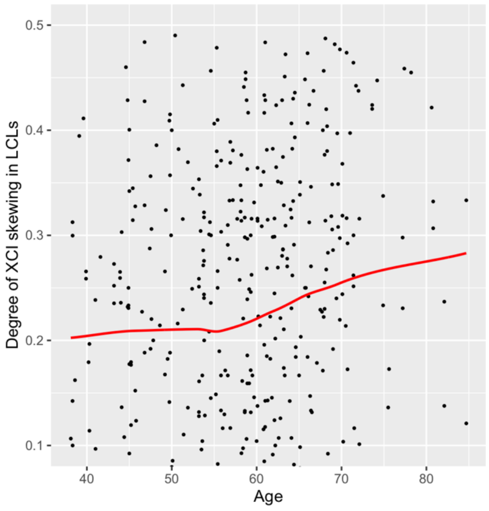

Supplementary Figure 6. Scatter plot of age (x-axis) and degree of XCI skewing (DS) in LCLs samples (y-axis). Ages range from 38 to 85 with a median age of 60 (Supplementary Figure 1). DS<0.3 indicates random XCI patterns while DS ≥0.3 indicates skewed XCI patterns. Each dot represents an individual. The red line represents the lowess curve between age and DS. Lowess curve detects an inflection point at approximately 55 years of age, after which DS starts to increase.

Supplementary Tables

Tissue [age group]	Monozygotic twin pairs	Dizygotic twin pairs	Total twin pairs
LCLs [<55]	18	25	43
LCLs [≥55]	43	38	81
Whole-blood [<55]	4	4	8
Whole-blood [≥55]	7	7	14
Fat [<55]	17	19	36
Fat [≥55]	34	45	79
Skin [<55]	17	17	34
Skin [≥55]	27	32	59

Supplementary Table 1. Number of monozygotic and dizygotic twin pairs with informative XCI calls for both co-twins in each tissue.

	Number of never smokers	Number of current Smokers	Total number
**All ages**	233	37	270
**< 55**	76	22	98
**≥ 55**	157	15	172

Supplementary Table 2. Sample size for smoking analysis. Younger (age < 55) and older (age ≥ 55) women were classified as never smokers or current smokers according to consistency in self-reported questionnaire data taken both at the time of sampling and 5.1 ± 0.70 years later[9]. Past smokers were excluded from analysis.

Supplementary References

1 Tukiainen, T., Villani, A. C., Yen, A., Rivas, M. A., Marshall, J. L., Satija, R., Aguirre, M., Gauthier, L., Fleharty, M., Kirby, A., Cummings, B. B., Castel, S. E., Karczewski, K. J., Aguet, F., Byrnes, A., Consortium, G. T., Laboratory, D. A., Coordinating Center -Analysis Working, G., Statistical Methods groups-Analysis Working, G., Enhancing, G. g., Fund, N. I. H. C., Nih/Nci, Nih/Nhgri, Nih/Nimh, Nih/Nida, Biospecimen Collection Source Site, N., Biospecimen Collection Source Site, R., Biospecimen Core Resource, V., Brain Bank Repository-University of Miami Brain Endowment, B., Leidos Biomedical-Project, M., Study, E., Genome Browser Data, I., Visualization, E. B. I., Genome Browser Data, I., Visualization-Ucsc Genomics Institute, U. o. C. S. C., Lappalainen, T., Regev, A., Ardlie, K. G., Hacohen, N. & MacArthur, D. G. Landscape of X chromosome inactivation across human tissues. *Nature* **550**, 244-248 (2017).

2 Shvetsova, E., Sofronova, A., Monajemi, R., Gagalova, K., Draisma, H. H. M., White, S. J., Santen, G. W. E., Chuva de Sousa Lopes, S. M., Heijmans, B. T., van Meurs, J., Jansen, R., Franke, L., Kielbasa, S. M., den Dunnen, J. T., t

Hoen, P. A. C., consortium, B. & Go, N. L. c. Skewed X-inactivation is common in the general female population. *Eur J Hum Genet* **27**, 455-465 (2019).
3 Brown, C. J., Ballabio, A., Rupert, J. L., Lafreniere, R. G., Grompe, M., Tonlorenzi, R. & Willard, H. F. A gene from the region of the human X inactivation centre is expressed exclusively from the inactive X chromosome. *Nature* **349**, 38-44 (1991).
4 Brown, C. J., Hendrich, B. D., Rupert, J. L., Lafreniere, R. G., Xing, Y., Lawrence, J. & Willard, H. F. The human XIST gene: analysis of a 17 kb inactive X-specific RNA that contains conserved repeats and is highly localized within the nucleus. *Cell* **71**, 527-542 (1992).
5 Rupert, J. L., Brown, C. J. & Willard, H. F. Direct detection of non-random X chromosome inactivation by use of a transcribed polymorphism in the XIST gene. *Eur J Hum Genet* **3**, 333-343 (1995).
6 Bolduc, V., Chagnon, P., Provost, S., Dube, M. P., Belisle, C., Gingras, M., Mollica, L. & Busque, L. No evidence that skewing of X chromosome inactivation patterns is transmitted to offspring in humans. *J Clin Invest* **118**, 333-341 (2008).
7 Amos-Landgraf, J. M., Cottle, A., Plenge, R. M., Friez, M., Schwartz, C. E., Longshore, J. & Willard, H. F. X chromosome-inactivation patterns of 1,005 phenotypically unaffected females. *Am J Hum Genet* **79**, 493-499 (2006).
8 O'Connell, J., Gurdasani, D., Delaneau, O., Pirastu, N., Ulivi, S., Cocca, M., Traglia, M., Huang, J., Huffman, J. E., Rudan, I., McQuillan, R., Fraser, R. M., Campbell, H., Polasek, O., Asiki, G., Ekoru, K., Hayward, C., Wright, A. F., Vitart, V., Navarro, P., Zagury, J. F., Wilson, J. F., Toniolo, D., Gasparini, P., Soranzo, N., Sandhu, M. S. & Marchini, J. A general approach for haplotype phasing across the full spectrum of relatedness. *PLoS Genet* **10**, e1004234 (2014).
9 Tsai, P. C., Glastonbury, C. A., Eliot, M. N., Bollepalli, S., Yet, I., Castillo-Fernandez, J. E., Carnero-Montoro, E., Hardiman, T., Martin, T. C., Vickers, A., Mangino, M., Ward, K., Pietilainen, K. H., Deloukas, P., Spector, T. D., Vinuela, A., Loucks, E. B., Ollikainen, M., Kelsey, K. T., Small, K. S. & Bell, J. T. Smoking induces coordinated DNA methylation and gene expression changes in adipose tissue with consequences for metabolic health. *Clin Epigenetics* **10**, 126 (2018).
